# Long-Term Burden and Respiratory Effects of Respiratory Syncytial Virus Hospitalization in Preterm Infants—The SPRING Study

**DOI:** 10.1371/journal.pone.0125422

**Published:** 2015-05-08

**Authors:** Xavier Carbonell-Estrany, Eduardo G. Pérez-Yarza, Laura Sanchez García, Juana M. Guzmán Cabañas, Elena Villarrubia Bòria, Belén Bernardo Atienza, IRIS (Infección Respiratoria Infantil por Virus Respiratorio Sincitial) Study Group

**Affiliations:** 1 Neonatology Service, Hospital Clinic, Institut d'Investigacions Biomediques August Pi Suñer (IDIBAPS), Barcelona, Spain; 2 Division of Pediatric Respiratory Medicine, Hospital Universitario Donostia—Instituto Biodonostia, San Sebastián, Spain; 3 Biomedical Research Centre Network for Respiratory Diseases (CIBERES), San Sebastián, Spain; 4 Department of Pediatrics, University of the Basque Country (UPV/EHU), San Sebastián, Spain; 5 Neonatology Unit, Hospital Universitario La Paz, Madrid, Spain; 6 Neonatology Service, Hospital Universitario Reina Sofía, Córdoba, Spain; 7 Health Outcomes Research Department, 3D Health Research, Barcelona, Spain; 8 Neonatology Division, Instituto de Investigación Sanitaria Gregorio Marañón, Hospital General Universitario “Gregorio Marañón”, Madrid, Spain; Kliniken der Stadt Köln gGmbH, GERMANY

## Abstract

The health status of premature infants born 32^1^-35^0^ weeks’ gestational age (wGA) hospitalized for RSV infection in the first year of life (cases; n = 125) was compared to that of premature infants not hospitalized for RSV (controls; n = 362) through 6 years. The primary endpoints were the percentage of children with wheezing between 2-6 years and lung function at 6 years of age. Secondary endpoints included quality of life, healthcare resource use, and allergic sensitization. A significantly higher proportion of cases than controls experienced recurrent wheezing through 6 years of age (46.7% vs. 27.4%; p = 0.001). The vast majority of lung function tests appeared normal at 6 years of age in both cohorts. In children with pulmonary function in the lower limit of normality (FEV_1_ Z-score [-2; -1]), wheezing was increased, particularly for cases vs. controls (72.7% vs. 18.9%, p = 0.002). Multivariate analysis revealed the most important factor for wheezing was RSV hospitalization. Quality of life on the respiratory subscale of the TAPQOL was significantly lower (p = 0.001) and healthcare resource utilization was significantly higher (p<0.001) in cases than controls. This study confirms RSV disease is associated with wheezing in 32-35 wGA infants through 6 years of age.

## Introduction

Acute lower respiratory tract infection (LRTI) caused by respiratory syncytial virus (RSV) is one of the most prevalent causes of hospitalization during infancy [1,2]. For premature infants, the risk of RSV hospitalization is considerably elevated, with estimates suggesting that somewhere between 4% and 10% of moderate to late preterm (32^1^–35^0^ weeks’ gestational age [wGA]) infants are hospitalised with RSV-LRTI in the first year of life [3,4,5,6,7]. In addition to the acute burden placed on pediatric services during the winter season, RSV hospitalisation has been associated with on-going respiratory morbidity characterised by transient early wheezing and recurrent wheezing [8,9,10,11,12,13,14,15], reduced pulmonary function [12,16,17], and a higher risk or predisposition to asthma and allergies [12,18,19,20,21,22,23,24]. Such longer-term respiratory morbidity may reduce quality of life and result in considerable health care costs [25,26].

The recently published MAKI trial has implicated severe RSV infection as an important mechanism in the pathogenesis of recurrent wheezing in the first year of life in preterm infants born 33–35 wGA [9]. Whilst this study confirms a link between RSV hospitalization and early wheeze [9], the longer term respiratory consequences of RSV hospitalization in these moderate preterm infants requires further elucidation. In our present multicentre, observational, nested, case-control study with independent cohorts (SPRING study), we assessed the impact of RSV hospitalization on future health status of premature infants born 32–35 wGA through 6 years of age.

## Materials and Methods

### Patients

Children were recruited from FLIP-2 [3], a prospective, 2-cohort study, undertaken to validate the risk factors for RSV-LRTI hospitalization in premature infants born at 32^1^–35^0^ wGA identified in the earlier, case-controlled, FLIP study.[27] FLIP-2 includes 5,441 children born between 2005 and 2006 in 37 Spanish hospitals, 202 of whom were hospitalized with RSV in the first 12 months of life [3]. To avoid sample bias, children who fulfilled all eligibility criteria were randomly selected and invited to participate in the study.

### RSV cohort

Cases were children born prematurely between 32^1^ and 35^0^ wGA who were hospitalized for RSV respiratory infection under the age of 12 months. RSV infection was confirmed by immunofluorescence, enzyme-linked immunosorbent assay, or viral culture (RT-PCR was not widely available in Spain at this time); no attempt was made to standardize RSV testing methodology. A respiratory infection attributable to RSV was defined as: a positive result from an RSV test performed on the child between 7 days before and 72 hours after admission.

### Non-RSV cohort

Controls were children born prematurely between 32^1^ and 35^0^ wGA who had no hospitalization for any acute respiratory illness during the RSV season and who were under the age of 12 months.

### Exclusion criteria for cases and controls

Medical charts were reviewed at study entry and children with any of the following were excluded from participation: diagnosis of chronic lung disease of prematurity or other chronic pulmonary diseases; diagnosis of hemodynamically significant congenital heart disease; congenital abnormalities of the airways; any neuromuscular disease; known immunodeficiency; any illness or condition that would preclude long-term survival; or participation in a trial of an investigational RSV prophylaxis or therapeutic agent. A diagnosis of asthma at 2 years of age was an exclusion criterion due to the lack of certainty of the diagnosis at this age and its potential as a confounding factor in the analysis.

### Ethics Statement

The study was conducted according to the principles of the 1964 Declaration of Helsinki and standards of Good Clinical Practice as specified in Circular Letter 15/2002 from the Spanish Drug Agency. The study was approved centrally by the Clinical Research Ethical Committee of the Hospital Clinic, Barcelona (Spain) (number: 2008/4468) and was also approved locally by the Clinical Research Ethical Committee of the Hospital General Universitario Gregorio Marañon, Madrid (Spain), the Clinical Research Ethical Committee of the Hospital Universitario Príncipe de Asturias, Alcala de Henares, Madrid (Spain), and the Clinical Research Ethical Committee of the Hospital de Jaén, Andalucía (Spain). The study is fully compliant with the Personal Data Protection Act. Parents/legal guardians provided written informed consent for all children included in the study.

### Study Design and Follow-up

**Fig 1:**
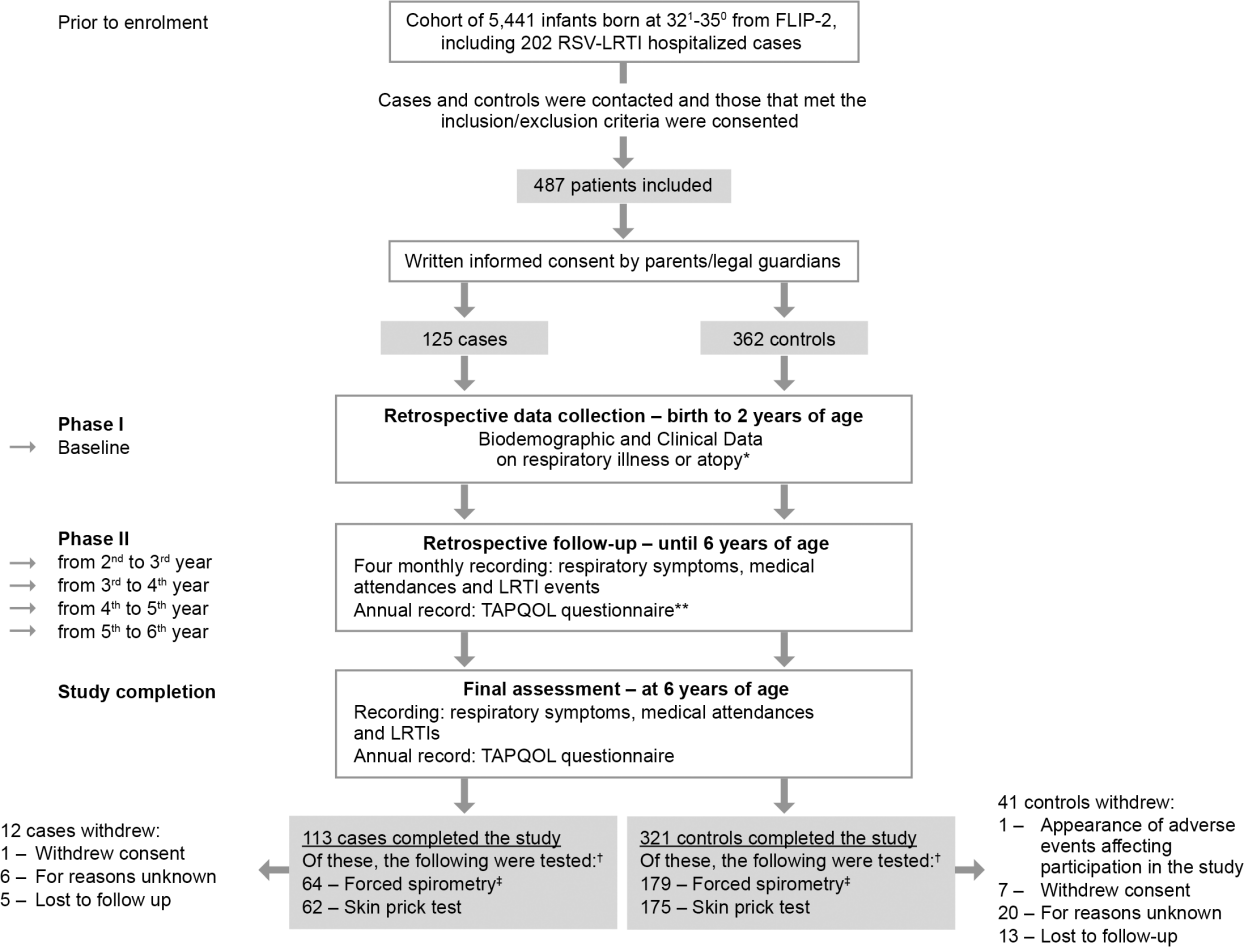
Schematic of Study Design. *children: allergic dermatitis, allergic rhinitis, allergic conjunctivitis, or contact dermatitis; parents/siblings: asthma, food allergy, pollen allergy, mite allergy, contact dermatitis, or allergic dermatitis. **TNO-AZL preschool children Quality of Life questionnaire: a 43-item questionnaire consisting of 12 multi (3–7) item scales that cover the domains of physical (sleeping, appetite, respiratory problems, digestive problems, skin problems, motor functioning), social (social functioning, problem behaviour), cognitive (communication), and emotional functioning (anxiety, positive mood, liveliness). Scale scores are calculated by adding up the item scores within the scales, and transforming the crude scale scores linearly to a 0–100 scale, with higher scores indicating better quality of life^28,29,30^. †Parents/legal guardians had to provide additional permissions for lung function and skin prick tests to be performed. ‡FEV_1_, FVC, FEF_25-75_.

The FLIP-2 study included prospective data collection of baseline characteristics and sociodemographics data for all infants during the first year of life (2005 to 2006 and 2006 to 2007) [3]. Depending on whether the child was born in 2005 or 2006, a further 2 or 1 years’ data on respiratory illness were collected retrospectively, covering the period from the FLIP-2 study completing (2006/2007) and the SPRING study starting in 2008 (Fig 1). The prospective surveillance period included telephone follow-up calls every four months and annual visits until the sixth year of life. Data on respiratory issues, including details of hospital attendances, admissions and treatment, were collected at each follow-up. Children’s quality of life was assessed on an annual basis using the validated Spanish version of the TNO-AZL Preschool children Quality of Life questionnaire (TAPQOL) [28,29,30]. At the end of the follow-up period, a test of pulmonary function by forced spirometry was performed in all children whose parents provided permission, with all hospitals following the same methodology in accordance with the American Thoracic Society (ATS) and the European Respiratory Society (ERS) 2005 standards [31]. To test whether atopy was a potential marker for wheezing, a skin prick test for immunoglobulin E (IgE)-mediated allergic reactivity was undertaken at the end of the follow-up period, using standard methodology [32,33]. The lung function and skin prick tests were performed only in children whose parents/legal guardians had provided additional consent for each individual test.

### Study Outcomes

The primary outcomes were the percentage of children with wheezing from age 2 to 6 and lung function at 6 years of age compared between cases and controls. Wheezing rates were analysed for each year of follow-up and across the whole study period (2 to 6 years). Wheezing reported either from parents/carers or physician was defined as follows:

**Simple wheezing**—defined as <3 episodes of wheezing within 12 months; where a wheezing episode constituted one or more days of wheezing preceded and followed by a healthy non-wheeze period of at least one week.

**Recurrent wheezing**—defined as three or more simple wheezing or bronchiolitis episodes within a year (i.e. at least 3 wheezing episodes within 12 months). Bronchiolitis was included in the definition to maximise capture of total respiratory morbidity.

**Severe recurrent wheezing**—defined as recurrent wheezing associated with at least one episode of hospitalization, or three or more medical attendances (outpatient, emergency or home), or one or more courses of systemic steroids, or asthma medication for at least three consecutive months or five cumulative months in a year.

**Total wheezing**—defined as those patients who had presented with any episode of wheezing, whether simple, recurrent, or severe.

Since a family history of atopy has been associated with an increased risk of recurrent wheezing [34], the incidence of wheezing was also analysed separately in those children with a diagnosis of an atopic condition (allergic rhinitis, allergic conjunctivitis or atopic skin) or in those where family atopy (including asthma, food allergy or cutaneous allergy) is present in a close family member (parents or siblings). As a result of being hospitalized for RSV, cases had potentially more medical attendances/contacts than controls in the first year of life; hence, to avoid the potential for bias conferred from any underreporting of respiratory morbidity for children in the control group, outcomes for wheezing were assessed from years 2 to 6 across both cohorts.

Lung function was assessed by mean values of spirometry parameters, including: FVC (forced vital capacity), FEV_1_ (forced expiratory volume in one second), and FEF_25-75_ (mean forced expiratory flow between 25% and 75% of FVC). Z-scores were calculated from FEV_1_ values and analyses were undertaken stratifying the results by wheezing history. Reference values for spirometry were taken from the paper by Quanjer et al [35] and Z-scores calculated using The Global Lungs Initiative equations freely available at www.lungfunction.org.

Secondary outcomes were: mean scores for each of the 12 scales of the TAPQOL questionnaire (Fig 1), healthcare resource use for respiratory illness through 2 to 6 years (hospital attendances, hospital admissions and treatment), and allergic sensitization compared between cases and controls.

### Statistical Analysis

The sample size calculation for this case-control study with independent cohorts was based on the rate of recurrent wheezing being 15% higher in the RSV hospitalized group than the not hospitalized group, which was assumed to have a rate of 16%.[22,34,36] To detect differences through a Chi-squared (χ²) test for two independent samples, it was estimated that the study would require 337 controls and 113 cases (proportion: approximately 3/1; to compensate for the expected difficulty in recruiting cases without compromising the statistical parameters); to achieve 0.05 significance (α) at 80% power (1-β), allowing for a 30% withdrawal rate over the 6 years of the study.

The Student’s t-test and Mann-Whitney *U* test (nonparametric statistical analyses) were used to compare continuous variables between cases and controls, and the χ^2^ test and Fisher Exact test for discrete variables. The Log-rank test evaluated changes in the percentage of children wheezing over time between groups. Outcomes for each year of follow-up included all available data for that particular year. For cumulative outcomes across years 2 to 6, all patient data from those years were included in the analyses, with any history of wheezing included as a wheezing event for those children with incomplete follow-up. A multivariate forward stepwise logistic regression model was developed to determine the relative contribution of any variable to any differences in wheezing between cases and controls through years 1 to 6. Adjusted odds ratios (ORs) were utilised to determine the relative contribution of each variable in the model. Comparisons of changes in the TAPQOL questionnaire (total score and subscale scores) used the Mann-Whitney *U* test.

All tests were carried out bilaterally (Fisher Exact test unilaterally), with a confidence level of 95%.

## Results

### Patients

A total of 125 cases and 362 controls were located, met the inclusion criteria, and were consented to be enrolled in the study (Fig 1). Four cases and 12 controls had received palivizumab and no children were excluded from the study on the basis of an asthma diagnosis by 2 years of age. The 125 cases were preterm infants born 32–35 wGA who were hospitalized for RSV respiratory infection in the first year of life (out of the 202 in the FLIP-2 study). The 362 controls were infants not hospitalized for RSV. The cases and controls were similar in terms of gestational age, sex, breast feeding history, number of residents at home, presence of animals at home, and family history of atopy (Table 1). However, mean birth weight (p = 0.008) was lower and there was more multiple pregnancy (p = 0.010) in the control group, whilst smoking (p = 0.010) and carpets at home (p = 0.005) were more frequent in cases. Of the 434 infants who completed 6 years of follow-up (113 cases and 321 controls), 276 underwent forced spirometry (243 accepted: 64 cases and 179 controls) and 236 skin prick test (62 cases and 174 controls) (Fig 1).

**Table 1:** Patient demographics and background characteristics.

Variable	Case (n = 125)	Control (n = 362)	*p* *
Age at study entry			NS
1 year, n (%)	5 (4.0%)	6 (1.7%)	
2 years, n (%)	53 (42.4%)	144 (39.8%)	
3 years, n (%)	67 (53.6%)	212 (58.6%)	
Gestational age, mean weeks (SD)	33.7 (0.92)	33.6 (0.81)	NS
32 weeks, n (%)	15 (12.0%)	36 (9.9%)	
33 weeks, n (%)	31 (24.8%)	98 (27.1%)	
34 weeks, n (%)	54 (43.2%)	188 (51.9)	
35 weeks, n (%)	25 (20.0%)	40 (11.1%)	
Birth weight, mean g (SD)	2098 (362)	1996 (377)	0.008
Male sex, n (%)	72 (57.6%)	203 (56.1%)	NS
White ethnicity, n (%)	110 (88.0%)	328 (90.6%)	NS
Multiple pregnancy, n (%)	53 (42.4%)	199 (55.0%)	0.010
Breast feeding			
n (%)^a^	57 (45.6%)	179 (49.4%)	NS
median months (P-25-P75)	3 (2–6)	3 (1–5)	NS
Residents at home, median n (P-25-P75)	3 (3–4)	3 (3–4)	NS
Smoking at home, n (%)	70 (56.0%)	157 (43.4%)	0.010
Animals in the home, n (%)^b^	26 (20.8%)	82 (22.7%)	NS
Carpets in the home, n (%)^b^	60 (48.0%)	126 (34.8%)	0.005
Family history of atopy^c^			
Close relative, n (%)	73 (58.4%)	189 (52.2%)	NS
First degree relative, n (%)	62 (49.6%)	166 (45.9%)	NS

SD: standard deviation, NS: not significant.

^a^5 missing values (2 for cases; 3 for controls).

^b^1 missing value for cases.

^c^close relative includes mother, father, siblings and other family; first degree relative includes only mother, father and siblings; atopy includes diagnosis of asthma, food allergy, mite allergy, contact dermatitis, or skin allergy.

*t-test, χ^2^ test, Mann-Whitney *U* test.

### Wheezing

Through 6 years of age, the incidence of wheezing (simple, recurrent, severe or total) was significantly higher in the RSV cohort than in control children (Table 2). Nearly half of cases (46.7%) had experienced recurrent wheezing by 6 years of age compared to around a quarter of controls (27.4%; p = 0.001), with the onset of recurrent wheezing occurring significantly earlier in the former than latter (mean years to onset: 4.69 vs. 5.29 years, respectively; p<0.001). The majority of recurrent wheezing recorded could be considered severe, as the rates were not much lower in the former than the latter over the 6 years of follow-up. Whilst the incidence of recurrent wheezing was higher in cases than controls for each individual year of follow-up, the difference remained statistically significant only until the children reached 3 years of age. When considering total wheezing, however, a significantly higher rate of wheezing was observed in cases than controls until children reached 5 years of age (32.5% vs. 24.0%, respectively; p = 0.047). In the sub-group of infants with severe wheezing, a statistically significant higher rate was observed in cases than controls at 2, 3 and 5 years, although not at 4 years. At 6 years of age, wheezing was not significantly different between cases and controls for any of the definitions (simple, recurrent, severe or total).

**Table 2:** Wheezing through 6 years of age^a^.

Wheezing	Year 2		Year 3		Year 4		Year 5		Year 6		Years 2–6	
	Case	Control	Case	Control	Case	Control	Case	Control	Case	Control	Case	Control
Simple wheezing												
n/N	24/70	46/181	44/123	88/358	32/124	54/350	27/120	58/333	18/113	44/321	80/120	174/354
(%)	(34.3)	(25.4)	(35.8)	(24.6)	(25.8)	(15.4)	(22.5)	(17.4)	(15.9)	(13.7)	(66.7)	(49.2)
*p* *	NS		0.012		0.008		NS		NS		0.001	
OR (95%CI)	1.53 (0.84–2.78)		1.71 (1.10–2.65)		1.91 (1.16–3.13)		1.38 (0.82–2.30)		1.19 (0.66–2.16)		2.07 (1.34–3.19)	
Recurrent wheezing												
n/N	29/70	22/182	36/123	55/358	23/124	44/350	18/120	31/333	14/113	31/321	35/75	95/347
(%)	(41.4)	(12.1)	(29.3)	(15.4)	(18.5)	(12.6)	(15.0)	(9.3)	(12.4)	(9.7)	(46.7)	(27.4)
*p* *	<0.001		0.001		NS		NS		NS		0.001	
OR (95%CI)	5.14 (2.68–9.87)		2.28 (1.41–3.70)		1.58 (0.91–2.75)		1.72 (0.92–3.20)		1.32 (0.68–2.59)		2.32 (1.39–3.87)	
Severe wheezing												
n/N	25/70	18/181	33/123	47/358	22/124	43/350	16/120	22/333	9/113	28/321	29/77	83/350
(%)	(35.7)	(9.9)	(26.8)	(13.1)	(17.7)	(12.3)	(13.3)	(6.6)	(8.0)	(8.7)	(37.7)	(23.7)
*p* *	<0.001		0.001		NS		0.021		NS		0.010	
OR (95%CI)	5.03 (2.52–10.0)		2.43 (1.47–4.01)		1.54 (0.88–2.70)		2.18 (1.10–4.30)		0.91 (0.41–1.98)		1.94 (1.15–3.28)	
Total wheezing												
n/N	42/70	63/181	66/123	126/358	47/124	91/350	39/120	80/333	26/113	66/321	50/70	186/342
(%)	(60.0)	(34.8)	(53.7)	(35.2)	(37.9)	(26.0)	(32.5)	(24.0)	(23.0)	(20.6)	(71.4)	(54.4)
*p* *	<0.001		<0.001		0.009		0.047		NS		0.006	
OR (95%CI)	2.81 (1.59–4.96)		2.12 (1.40–3.22)		1.74 (1.13–2.68)		1.52 (0.96–2.41)		1.16 (0.69–1.93)		2.10 (1.20–3.67)	

CI: confidence interval, OR: odds ratio, NS: not significant.

^a^For each individual year of follow-up, the number (proportion) of children with wheezing is shown, utilising all available data for that particular year (thus, the same child could be included in more than one year). For cumulative outcomes across years 2 to 6, the number (proportion) of children with wheezing during those years is shown, with any history of wheezing included as a wheezing event for those children with incomplete follow-up (thus, each child is counted a maximum of once for each type of wheeze). It was not possible to classify every child into each type of wheezing (simple, recurrent, severe, total), which is reflected in the respective sample sizes.

*χ^2^ test.

A history of family atopy or childhood atopy did not appear to influence rates of wheezing (simple, recurrent, severe or total) through 6 years of age (S1 Table). In children with a diagnosis of an atopic condition, the incidence of wheezing was significantly higher in cases than controls throughout the study period (simple wheezing, 2–6 years: 67.4% vs. 49.8%, respectively, p<0.004; recurrent: 54.9% vs. 26.8%, p<0.001; severe: 45.3% vs. 24.3%, p = 0.003) (S2 Table).

Multivariate logistic regression analysis demonstrated that hospitalization with RSV in the first year of life was the most significant independent risk factor for wheezing during years 1 to 6 of childhood (Table 3). Other independent risk factors identified included having carpets in the home (for all types of wheezing), birth weight (only for recurrent wheezing), and having a diagnosis of an atopic condition (only for severe wheezing).

**Table 3:** Logistic regression analysis* of risk factors associated with wheezing through 1–6 years.

	Risk Factors	Coefficients (SE)	OR (95%CI)	*p*
**Simple wheezing**	RSV hospitalization	0.713 (0.224)	2.04 (1.32–3.17)	0.001
	Carpets in the home	0.406 (0.195)	1.50 (1.03–2.12)	0.037
**Recurrent wheezing**	RSV hospitalization	1.482 (0.227)	4.40 (2.82–6.86)	<0.001
	Carpets in the home	0.526 (0.203)	1.69 (1.14–2.52)	0.009
	Birth weight	0.001 (0.000)	1.001 (1.000–1.001)	0.032
**Severe wheezing**	RSV hospitalization	1.461 (0.223)	4.31 (2.78–6.68)	<0.001
	Diagnosed with atopy 0–6 years	0.516 (0.205)	1.68 (1.12–2.50)	0.012
	Carpets in the home	0.411 (0.207)	1.51 (1.01–2.27)	0.047
**Total wheezing**	RSV hospitalization	1.316 (0.268)	3.73 (2.21–6.31)	<0.001
	Carpets in the home	0.544 (0.208)	1.72 (1.15–2.59)	0.009

CI: confidence interval, OR: odds ratio.

*Significant (p<0.05) variables in bivariate analyses were included in the forward, stepwise, logistic regression model. The following variables were included in the bivariate analyses: RSV hospitalization; sex; gestational age; multiple pregnancy; birth weight; breast fed; residents at home; smoking at home; animals in the home; carpets in the home; family history of atopy in first degree relative (mother, father and siblings); children with atopy diagnosed between 0–6 years (S5 Table).

### Lung Function

At 6 years of age, there were no statistically significant differences between cases and controls in baseline FEV_1_, FVC or FEF_25-75_ for absolute values or Z-score versus control values, in forced spirometry (S3 Table). For those children with a FEV_1_ Z-score [-2; -1] (11 of 64 cases [17.2%] and 37 of 179 controls [20.7%]), a statistically significant greater proportion of cases than controls had a history of simple, recurrent or severe wheezing (Table 4). The rates of wheezing (simple, recurrent and severe) were higher for this subpopulation of cases than in the overall case population, but rates were similar for controls. A statistically significant greater proportion of cases than controls also had recurrent, severe or total wheezing for FEV_1_ Z-score [-1; +1] (all p<0.001) and [+1; +2] (all p<0.05); there was no difference for simple wheezing for either ranking. When the cases and controls were analysed separately, there were no differences in the incidence of wheezing (simple, recurrent, severe or total) in either group for total, [-2; -1], [-1; +1] or [+1; +2] FEV_1_ Z-score.

**Table 4:** Respiratory function Z-score^a^ FEV_1_ ranking [-2; -1] stratified by wheezing.

	Case (n = 11)	Control (n = 37)	*p* *	OR (95%CI)
Simple wheezing, n (%)	10 (90.9)	19 (51.4)	0.018	9.47 (1.10–81.68)
Recurrent wheezing, n (%)	8 (72.7)	7 (18.9)	0.002	11.43 (2.40–54.45)
Severe wheezing, n (%)	6 (54.5)	6 (16.2)	0.018	6.20 (1.42–27.07)

CI: confidence interval, OR: odds ratio, NS: not significant. FEV_1_ in the extreme range Z-score (-1, -2), cases have increased respiratory morbidity (simple, recurrent and severe wheezing) in comparison with controls.

^a^Z-score: the number of standard deviations that the observed FEV_1_ deviates from the predicted value from the reference population of healthy children. A value below -1.64 signifies a 95% chance of the value being outside the normal reference range.

*χ2 test.

### Quality of Life

**Fig 2:**
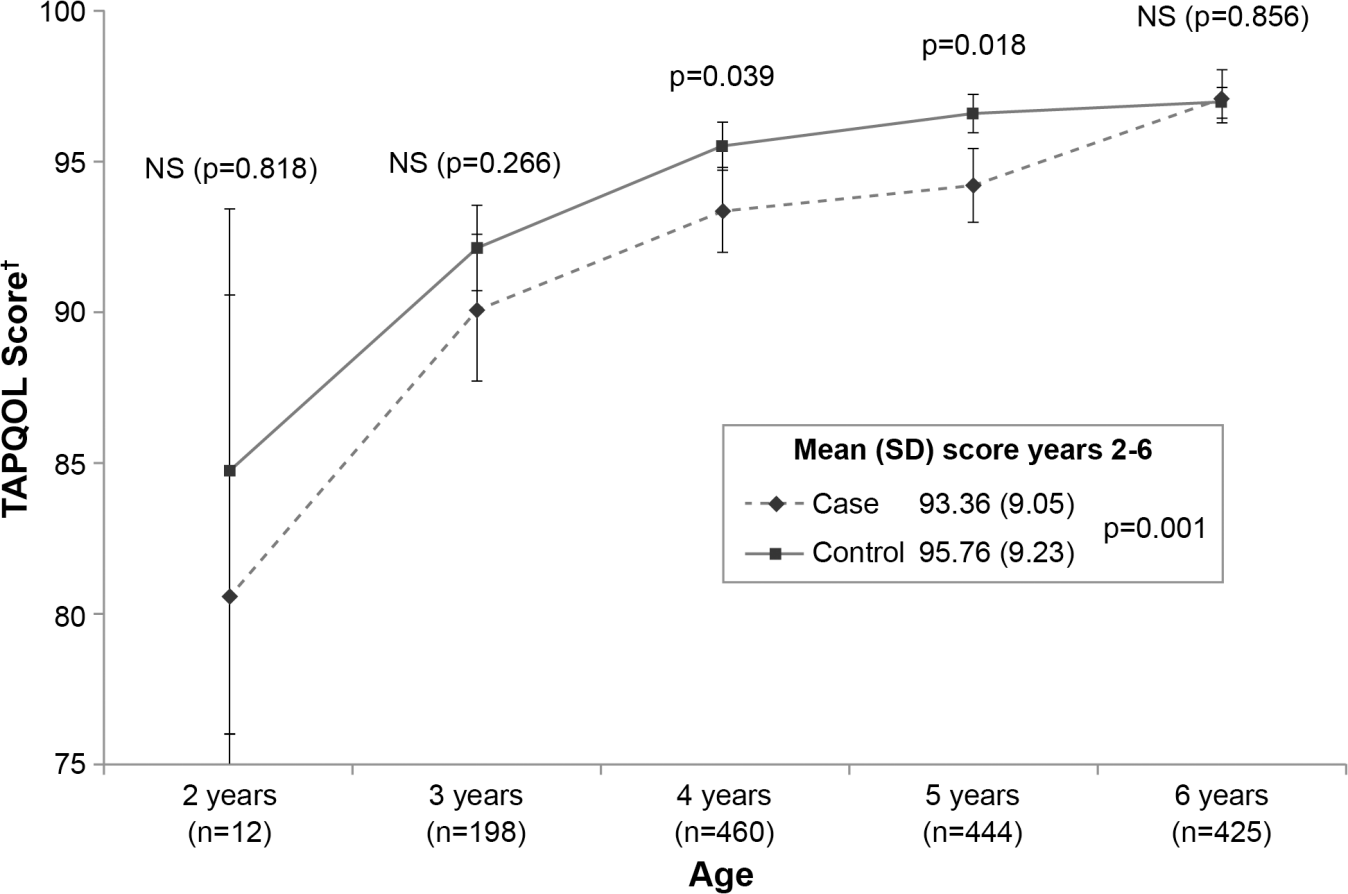
Respiratory scores for TAPQOL questionnaire through 6 years of age. †Scores range from 0–100, where higher scores indicate better quality of life for patients. Mann-Whitney *U* test. SD: standard deviation, NS: not significant. Patient numbers at years 2 and 3 reflect patients entering the study at different ages. For each patient, the mean score was calculated across the years they completed the questionnaire (e.g. if TAPQOL completed from 2 to 6 years, mean was calculated over 5 years).

Of the 12 domains constituting the TAPQOL questionnaire, only the score for the ‘respiratory problems’ dimension was significantly different between cases and controls through 6 years of age. The mean score on the respiratory problems scale of quality of life, throughout the first 6 years of life, was significantly lower in cases than controls (93.96 [SD 9.05] vs. 95.76 [9.23], respectively; p = 0.001). Quality of life on the respiratory problems scale was lower in cases than controls from 2 to 5 years of age, but the differences were statistically significant only at 4 and 5 years of age (p = 0.039 and p = 0.018, respectively) (Fig 2). At 6 years of age, quality of life for respiratory problems between cases and controls was similar.

### Healthcare Resource Use

A statistically significant greater proportion of cases than controls required outpatient care (84.0% vs. 66.3%, respectively; p<0.001) and emergency care (62.4% vs. 33.7%; p<0.001) for respiratory problems through 6 years of age (Table 5). The difference between cases and controls was statistically significant every individual year until children were 5 years of age for outpatient care and 4 years of age for emergency care. The percentage of children requiring asthma medications (bronchodilators, leukotriene antagonists, inhaled steroids, oral steroids) and antibiotics was significantly higher for cases than controls (Table 5).

**Table 5:** Hospital attendances, admissions and treatment for respiratory issues through 6 years of age.

Variable	Case (n = 125)	Control (n = 362)	*p* *
Outpatient care, n (%)	105 (84.0)	240 (66.3)	<0.001
Emergency care, n (%)	78 (62.4)	122 (33.7)	<0.001
Hospital admission, n (%)	12 (9.6)	23 (6.4)	NS
Bronchodilators, n (%)	78 (62.4)	153 (42.3)	<0.001
Leukotriene antagonists, n (%)	20 (16.0)	23 (6.4)	0.002
Inhaled steroids, n (%)	33 (26.4)	58 (16.0)	0.009
Antibiotics, n (%)	47 (37.6)	97 (26.8)	0.016
Oral steroids, n (%)	23 (18.4)	39 (10.8)	0.023

NS: not significant.

*χ^2^ test.

### Allergic Sensitization

At 3 and 4 years of age, the incidence of allergic rhinitis was significantly higher in cases than controls (3 years: 6.5% vs. 1.7%, respectively, p = 0.011; 4 years: 7.3% vs. 3.1%, p<0.05); rates were similar from 5 years of age onwards. The incidence of contact dermatitis was significantly higher in cases than controls at 3 (8.1% vs. 2.5%; p = 0.009) and 5 (7.5% vs. 3.0%; p = 0.038) years of age, but not at 4 or 6 years of age. There were no statistically significant differences between the groups regarding the incidence of skin allergies or allergic conjunctivitis at any age. The results of the skin prick test showed a statistically significant higher rate of positivity to dust mites in controls than cases (13.7% vs. 4.8%, respectively; p = 0.042) and a statistically significant higher rate of positivity to cats in cases than controls (6.5% vs. 1.1%, respectively; p = 0.043) (S4 Table). There were no statistically significant differences between the two groups for any of the other allergens tested.

## Discussion

The SPRING study has confirmed that severe RSV respiratory disease in moderate to late preterm infants (32–35 wGA) is linked to recurrent wheezing in early childhood. Results from this study showed recurrent wheezing and severe wheezing occurring in a significantly higher proportion of cases (40–50%) than controls (20–30%) by 6 years of age. These results are in line with previous studies involving moderate preterm infants that have shown an association between RSV infection and early childhood wheezing [9,13,34,37]. In a retrospective cohort study using linked inpatient, outpatient, and laboratory databases undertaken in the US, Escobar et al [13] reported that approximately 30–60% of premature infants 32–36 wGA with RSV infection during the first year of life will have a recurrent wheezing episode by 5 years of age. Other studies have shown RSV-associated wheezing at 1 year [9], 2 years [34], and 3 years [37] of age in moderate preterm infants. Our study has shown that the incidence of wheezing was consistently higher for cases than controls in every year group throughout the first 6 years of life, though the differences were statistically significant only at 2 and 3 years of age for recurrent wheezing, and 2, 3 and 5 years of age for severe wheezing. It is not known why the statistical significance varied between years, but it could possibly reflect yearly variations in the incidence and severity of respiratory diseases and may be other variables that we have not been able to control in this study.

Consistent with the results of a recent systematic review and meta-analysis of 15 studies exploring the link between RSV infection with recurrent wheezing and asthma [38], the SPRING study shows a decline in the incidence of wheezing with age. Interestingly though, other longer-term studies have shown the relationship between RSV infection and recurrent wheezing/asthma persisting for longer than the 5 years for severe wheezing seen in the SPRING study. In the studies by Henderson et al [22] and Sigurs et al [21] an increased prevalence of asthma/wheezing for RSV infected children remained apparent at 7 and 18 years’ follow-up, respectively. The study by Stein et al [23] indicated an increased incidence of wheeze in RSV infected children at 11 years of age, but that this difference disappeared at 13 years of age. Further evidence for the longer duration of RSV-attributable asthma comes from a recent systematic review of 28 studies that reported a similar level of risk for children ≤5 years (13–22%) to those aged 5 to 11 years (11–27%), with no apparent reduction in risk for children ≥12 years (32%) [39]. Differences in methodology, definitions of wheezing/asthma and patient populations, and non-focussing on moderate to late preterm infants [21,22,23], might be potential reasons for the shorter duration of wheezing seen in the SPRING study. Moreover, recent studies have also shown that genetic variations might influence the risk and severity of RSV infection and predisposition to recurrent wheezing [40,41].

Another potential factor that might influence respiratory morbidity is whether the child had an atopic diagnosis and/or a family history of atopy, since this has been shown to increase the risk of recurrent wheezing in preterm infants [34]. When analysed across the whole SPRING cohort, however, the atopic status (based on diagnosis *in probandus* or a family history of atopy) did not appear to significantly influence the rate of wheezing observed. This negative finding may be due to the age of the study population (6 years), as other studies have shown that atopy tends to be associated with later onset wheezing, rather than transient early wheezing [42,43]. With longer follow-up, atopy might become a more important variable in the SPRING population, as it was shown to play a role in severe wheezing. It has also been suggested that an atopic background might be such a strong predisposing factor for later wheezing that RSV hospitalization does not increase that risk very much [44]. This concurs with the results of the MAKI trial, which found that RSV prevention was associated with reduced wheezing in the first year of life, regardless of whether there was a family history of atopy [9]. It should also be recognised that the design of the SPRING study might not have been powered to detect any difference relating to atopic status. Overall, the single most important factor associated with wheezing in the SPRING study was whether children were hospitalized with RSV or not in the first year of life, as this made by far the largest contribution to the multivariate logistic regression analysis undertaken.

Increasing evidence suggests that preterm birth affects lung function. Indeed, prematurity has been shown to be independently associated with reduced lung function in the first months of life, with male sex, lower gestational age, and weight being important predictors for reduced flows in this group [45]. As well as short-term reductions in flows reported during the first months of life [45], %FEV_1_ has been shown to be decreased later in life (range 5–23 years) in preterm-born individuals, even in those who do not develop BPD [46]. Other studies have shown that premature birth, in the absence of neonatal respiratory disease, results in abnormal growth and development of the lung [47]. As a result of this abnormality, there are a persistently reduced flows and the absence of catch-up growth in airway function [47]. Whilst the overall results from the SPRING study showed normality in lung function, there was an important subgroup of cases and controls with pulmonary flow in the lower limit of normality at 6 years of age. Interestingly, our results showed that the premature subgroup with reduced flows (FEV_1_ Z-score [-2; -1]) had increased respiratory morbidity and that this is greater in those who were hospitalized versus non-hospitalized, which is consistent with the results reported by Escobar et al [16].

The clinical relevance and impact of childhood wheezing following RSV hospitalization was validated by the TAPQOL questionnaire, which showed a statistically significant decrease in health-related quality of life through 6 years of age for cases compared to controls, but only for the respiratory sub-score. The increase in the incidence of wheezing in RSV hospitalized infants was also associated with a commensurate increase in healthcare resource use for respiratory issues, both in terms of medical contacts (outpatient and emergency care) and treatments received (asthma medication and antibiotics). Pertinently, it was found that the majority of recurrent wheezing was reported as severe. Taken together, the long-term sequelae of severe RSV disease demonstrated in this study highlights the importance of preventative measures in terms of modification of known risk factors (e.g. parental smoking) and the use of palivizumab prophylaxis programs [3,4,34,37,48]. An increasing number of studies have shown that palivizumab prophylaxis is not only effective at reducing RSV hospitalizations in moderate preterm infants [3,48], but also at reducing subsequent respiratory morbidity [9,34,37].

The main limitation of the SPRING study was the necessity to capture 1–2 years’ data on respiratory morbidity retrospectively, covering the period from when the FLIP-2 study ended and the SPRING study began. It is acknowledged by the authors that gathering these data proved to be more difficult for the controls than cases, which might have resulted in an under reporting of respiratory morbidity for the former. For this reason, data from the second year of life were excluded from all analyses. Since physician confirmed wheezing was not captured uniformly at all sites, a less stringent definition of ‘parents/carers or physician’ reported wheezing had to be used. The study of lung function by forced spirometry has the limitations of this technique (in that it requires active collaboration by the patient) and was chosen because it is routinely used in clinical practice. With other methodologies, such as total body plethysmography or measurement of resistance by impulse oscillometry, the results could be different. However, these methods were not available to all researchers, which is a frequent limitation in multicenter studies. Another potential limitation is the number of children completing the lung function and allergic sensitization tests due to the requirement for additional consent for these procedures from parents. In any case, of the total study cohort (434 children), 56% (243 for forced spirometry) and 54% (236 for skin prick) completed these tests, which provided sufficient data for analysis. We also recognize as a methodological limitation the fact that lung function was measured only once, at the end of the 6 year follow-up period, and not repeatedly throughout the study. Nevertheless, the results do not differ from those reported by other authors [49].

## Conclusions

The SPRING study confirms that elevated levels of wheezing associated with severe RSV disease persist to at least 5 years in late preterm children, with resultant decreases in respiratory quality of life and increased healthcare resource utilization. Whilst the results of the lung function test showed overall normality, a subgroup was identified in the lower limit of normality which had significant respiratory morbidity.

## Supporting Information

S1 Table Wheezing through 6 years of age in children with and without an atopic history†. †Defined as children with a diagnosis of allergic dermatitis, allergic rhinitis, allergic conjunctivitis, or contact dermatitis, or parents/siblings with a diagnosis of asthma, food allergy, pollen allergy, mite allergy, contact dermatitis, or allergic dermatitis. Of the 487 patients, 321 had an atopic status (233 controls; 88 cases) and 166 did not (129 controls; 37 cases). For cumulative outcomes across years 2 to 6, all patient data from those years were included in the analyses, with any history of wheezing included as a wheezing event for those children with incomplete follow-up; it was not possible to classify every patient into each type of wheeze (simple, recurrent, severe, total), which is reflected in the respective sample sizes. *χ^2^ test. CI: confidence interval, OR: odds ratio, NS: not significant.
(DOCX)

S2 Table Wheezing through 6 years of age in children with an atopic history†. †Defined as children with a diagnosis of allergic dermatitis, allergic rhinitis, allergic conjunctivitis, or contact dermatitis, or parents/siblings with a diagnosis of asthma, food allergy, pollen allergy, mite allergy, contact dermatitis, or allergic dermatitis. For cumulative outcomes across years 2 to 6, all patient data from those years were included in the analyses, with any history of wheezing included as a wheezing event for those children with incomplete follow-up; it was not possible to classify every patient into each type of wheeze (simple, recurrent, severe, total), which is reflected in the respective sample sizes. *χ^2^ test. CI: confidence interval, OR: odds ratio, NS: not significant.
(DOCX)

S3 Table Baseline results for forced spirometry. FVC: forced vital capacity, FEV_1_: forced expiratory volume in one second, FEF_25-75_: mean forced expiratory flow between 25% and 75% of FVC. Patient numbers reflect successful performance for each parameter.
(DOCX)

S4 Table Prick test results. These antigens are the standard ones used in Spain. *χ^2^ test or Fisher's exact test. NS: not significant.
(DOCX)

S5 Table Bivariate analysis of risk factors associated with wheezing through 6 years. CI: confidence interval, OR: odds ratio. *χ^2^ test, Fisher's exact test, t-test, Mann-Whitney *U* test. †Mother, father and siblings. NS: not significant.
(DOCX)
