# Supplementary material for: Long-Term Burden and Respiratory Effects of Respiratory Syncytial Virus Hospitalization in Preterm Infants—The SPRING Study
Source: PLoS One. 2015 May 8;10(5):e0125422. doi: 10.1371/journal.pone.0125422 (PMC4425575; doi:10.1371/journal.pone.0125422)
Supplement: S1 Table — †Defined as children with a diagnosis of allergic dermatitis, allergic rhinitis, allergic conjunctivitis, or contact dermatitis, or parents/siblings with a diagnosis of asthma, food allergy, pollen allergy, mite allergy, contact dermatitis, or allergic dermatitis. Of the 487 patients, 321 had an atopic status (233 controls; 88 cases) and 166 did not (129 controls; 37 cases). For cumulative outcomes across years 2 to 6, all patient data from those years were included in the analyses, with any history of wheezing included as a wheezing event for those children with incomplete follow-up; it was not possible to classify every patient into each type of wheeze (simple, recurrent, severe, total), which is reflected in the respective sample sizes. *χ2 test. CI: confidence interval, OR: odds ratio, NS: not significant. (DOCX) [file pone.0125422.s001.docx]

**S1 Table. Wheezing through 6 years of age in children with and without an atopic history†**

| **Wheezing** | **Year 2** | | **Year 3** | | **Year 4** | | **Year 5** | | **Year 6** | | **Years 2-6** | |
| --- | --- | --- | --- | --- | --- | --- | --- | --- | --- | --- | --- | --- |
|  | Atopy | No atopy | Atopy | No atopy | Atopy | No atopy | Atopy | No atopy | Atopy | No atopy | Atopy | No atopy |
| Simple wheezing, n/N (%)  *p**  OR (95%CI) | 38/153  (24.8) | 32/98  (32.7) | 82/319  (25.7) | 50/162  (30.9) | 61/316  (19.3) | 25/158  (15.8) | 61/302  (20.2) | 24/151  (15.9) | 48/293  (16.4) | 14/141  (9.9) | 171/313  (54.6) | 83/161  (51.6) |
|  | NS  0.68 (0.39-1.19) | | NS  0.78 (0.51-1.78) | | NS  1.27 (0.76-2.12) | | NS  1.34 (0.76-2.25) | | 0.047  1.78 (0.94-3.47) | | NS  1.13 (0.77-1.66) | |
| Recurrent wheezing, n/N (%)  *p**  OR (95%CI) | 34/154  (22.1) | 17/98 (17.3) | 68/319  (21.3) | 23/162  (14.2) | 51/316  (16.1) | 16/158  (10.1) | 38/302  (12.6) | 11/151  (7.3) | 38/293  (13.0) | 7/141  (5.0) | 89/279  (31.9) | 41/143  (28.7) |
|  | NS  1.35 (0.71-2.58) | | 0.037  1.64 (0.98-2.74) | | 0.049  1.71 (0.94-3.10) | | NS  1.83 (0.91-3.70) | | 0.006  2.85 (1.24-6.56) | | NS  1.17 (0.75-1.81) | |
| Severe wheezing, n/N (%)  *p**  OR (95%CI) | 29/153  (19.0) | 14/98  (14.3) | 63/319  (19.7) | 17/162  (10.5) | 50/316  (15.8) | 15/158  (9.5) | 31/302  (10.3) | 7/151  (4.6) | 31/293  (10.6) | 6/141  (4.3) | 80/283  (28.3) | 32/144  (22.2) |
|  | NS  1.40 (0.70-2.81) | | 0.006  2.10 (1.18-3.72) | | 0.038  1.79 (0.97-3.30) | | 0.028  2.35 (1.01-5.48) | | 0.018  2.66 (1.08-6.54) | | NS  1.38 (0.86-2.21) | |
| Total wheezing,  n/N (%)  *p**  OR (95%CI) | 64/153  (41.8) | 41/98  (41.8) | 128/319  (40.1) | 64/162  (39.5) | 98/316  (31.0) | 40/158  (25.3) | 89/302  (29.5) | 30/151  (19.9) | 73/293  (24.9) | 19/141  (13.5) | 162/274  (59.1) | 74/138  (53.6) |
|  | NS  1.00 (0.60-1.67) | | NS  1.03 (0.70-1.51) | | NS  1.33 (0.86-2.04) | | 0.018  1.69 (1.05-2.70) | | 0.004  2.13 (1.23-3.70) | | NS  1.25 (0.83-1.89) | |

†Defined as children with a diagnosis of allergic dermatitis, allergic rhinitis, allergic conjunctivitis, or contact dermatitis, or parents/siblings with a diagnosis of asthma, food allergy, pollen allergy, mite allergy, contact dermatitis, or allergic dermatitis. Of the 487 patients, 321 had an atopic status (233 controls; 88 cases) and 166 did not (129 controls; 37 cases). For cumulative outcomes across years 2 to 6, all patient data from those years were included in the analyses, with any history of wheezing included as a wheezing event for those children with incomplete follow-up; it was not possible to classify every patient into each type of wheeze (simple, recurrent, severe, total), which is reflected in the respective sample sizes

*χ^2^ test

CI: confidence interval, OR: odds ratio, NS: not significant
