# Supplementary material for: Long-Term Burden and Respiratory Effects of Respiratory Syncytial Virus Hospitalization in Preterm Infants—The SPRING Study
Source: PLoS One. 2015 May 8;10(5):e0125422. doi: 10.1371/journal.pone.0125422 (PMC4425575; doi:10.1371/journal.pone.0125422)
Supplement: S2 Table — †Defined as children with a diagnosis of allergic dermatitis, allergic rhinitis, allergic conjunctivitis, or contact dermatitis, or parents/siblings with a diagnosis of asthma, food allergy, pollen allergy, mite allergy, contact dermatitis, or allergic dermatitis. For cumulative outcomes across years 2 to 6, all patient data from those years were included in the analyses, with any history of wheezing included as a wheezing event for those children with incomplete follow-up; it was not possible to classify every patient into each type of wheeze (simple, recurrent, severe, total), which is reflected in the respective sample sizes. *χ2 test. CI: confidence interval, OR: odds ratio, NS: not significant. (DOCX) [file pone.0125422.s002.docx]

**S2 Table. Wheezing through 6 years of age in children with an atopic history†**

| **Wheezing** | **Year 2** | | **Year 3** | | **Year 4** | | **Year 5** | | **Year 6** | | **Years 2-6** | |
| --- | --- | --- | --- | --- | --- | --- | --- | --- | --- | --- | --- | --- |
|  | Case | Control | Case | Control | Case | Control | Case | Control | Case | Control | Case | Control |
| Simple wheezing, n/N (%)  *p**  OR (95%CI) | 16/47  (34.0) | 22/106  (20.8) | 30/87  (34.5) | 52/232  (22.4 | 27/88  (30.7) | 34/228  (14.9) | 22/85  (25.9) | 39/217  (18.0) | 16/82  (19.5) | 32/211  (15.2) | 58/86  (67.4) | 113/227  (49.8) |
|  | NS  1.97 (0.69-4.23) | | 0.021  1.82 (1.06-3.12) | | 0.002  2.53 (1.41-4.52) | | NS  1.59 (0.88-2.89) | | NS  1.36 (0.70-2.63) | | 0.004  2.09 (1.24-3.52) | |
| Recurrent wheezing, n/N (%)  *p**  OR (95%CI) | 23/47  (48.9) | 11/107  (10.3) | 29/87  (33.3) | 39/232  (16.8) | 19/88  (21.6) | 32/228  (14.0) | 15/85  (17.6) | 23/217  (10.6) | 14/82  (17.1) | 24/211  (11.4) | 28/51  (54.9) | 61/228  (26.8) |
|  | <0.001  8.36 (3.59-19.50) | | 0.001  2.47 (1.41-4.35) | | NS  1.69 (0.90-3.17) | | NS  1.81 (0.89-3.66) | | NS  1.60 (0.79-3.28) | | <0.001  3.33 (1.79-6.22) | |
| Severe wheezing, n/N (%)  *p**  OR (95%CI) | 20/47  (42.6) | 9/106  (8.5) | 28/87  (32.2) | 35/232  (15.1) | 18/88  (20.5) | 32/228  (14.0) | 13/85  (15.3) | 18/217  (8.3) | 9/82  (11.0) | 22/211  (10.4) | 24/53  (45.3) | 56/230  (24.3) |
|  | <0.001  7.98 (3.26-19.5) | | 0.001  2.67 (1.50-4.75) | | NS  1.58 (0.83-2.98) | | NS  2.00 (0.93-4.28) | | NS  1.06 (0.47-2.41) | | 0.003  2.57 (1.39-4.78) | |
| Total wheezing,  n/N (%)  *p**  OR (95%CI) | 31/47  (66.0) | 33/106  (31.1) | 48/87  (55.2) | 80/232  (34.5) | 38/88  (43.2) | 60/228  (26.3) | 32/85  (37.6) | 57/217  (26.3) | 24/82  (29.3) | 49/211  (23.2) | 38/49  (77.6) | 124/225  (55.1) |
|  | <0.001  4.29 (2.07-8.90) | | 0.001  2.34 (1.42-3.86) | | 0.003  2.13 (1.27-3.56) | | 0.036  1.70 (1.00-2.89) | | NS  1.37 (0.77-2.43) | | 0.003  2.81 (1.37-5.78) | |

†Defined as children with a diagnosis of allergic dermatitis, allergic rhinitis, allergic conjunctivitis, or contact dermatitis, or parents/siblings with a diagnosis of asthma, food allergy, pollen allergy, mite allergy, contact dermatitis, or allergic dermatitis. For cumulative outcomes across years 2 to 6, all patient data from those years were included in the analyses, with any history of wheezing included as a wheezing event for those children with incomplete follow-up; it was not possible to classify every patient into each type of wheeze (simple, recurrent, severe, total), which is reflected in the respective sample sizes

*χ^2^ test

CI: confidence interval, OR: odds ratio, NS: not significant
