# Supplementary material for: Long-Term Burden and Respiratory Effects of Respiratory Syncytial Virus Hospitalization in Preterm Infants—The SPRING Study
Source: PLoS One. 2015 May 8;10(5):e0125422. doi: 10.1371/journal.pone.0125422 (PMC4425575; doi:10.1371/journal.pone.0125422)
Supplement: S3 Table — FVC: forced vital capacity, FEV1: forced expiratory volume in one second, FEF25-75: mean forced expiratory flow between 25% and 75% of FVC. Patient numbers reflect successful performance for each parameter. (DOCX) [file pone.0125422.s003.docx]

**S3 Table. Baseline results for forced spirometry**

| **Rank** | **FVC** | | **FEV_1_** | | **FEF_25-75_** | |
| --- | --- | --- | --- | --- | --- | --- |
|  | **Case**  **(n=56)** | **Control**  **(n=158)** | **Case**  **(n=58)** | **Control**  **(n=164)** | **Case**  **(n=53)** | **Control**  **(n=161)** |
| [-2, -1.5], n (%) | 6 (10.7) | 12 (7.6) | 3 (5.2) | 19 (11.6) | 3 (5.7) | 17 (10.6) |
| [-1.5, -1], n (%) | 7 (12.5) | 27 (17.1) | 8 (13.8) | 18 (11.0) | 11 (20.8) | 21 (13.0) |
| [-1, -0.5], n (%) | 10 (17.9) | 19 (12.0) | 13 (22.4) | 37 (22.6) | 13 (24.5) | 47 (29.2) |
| [-0.5, 0], n (%) | 10 (17.9) | 34 (21.5) | 11 (19.0) | 25 (15.2) | 11 (20.8) | 27 (16.8) |
| [0, 0.5], n (%) | 10 (17.9) | 31 (19.6) | 13 (22.4) | 33 (20.1) | 6 (11.3) | 27 (16.8) |
| [0.5, 1], n (%) | 5 (8.9) | 18 (11.4) | 4 (6.9) | 19 (11.6) | 8 (15.1) | 14 (8.7) |
| [1, 1.5], n (%) | 5 (8.9) | 10 (6.3) | 5 (8.6) | 10 (6.1) | 1 (1.9) | 4 (2.5) |
| [1.5, 2], n (%) | 3 (5.4) | 7 (4.4) | 1 (1.7) | 3 (1.8) | 0 (0.0) | 4 (2.5) |

FVC: forced vital capacity, FEV_1_: forced expiratory volume in one second, FEF_25-75_: mean forced expiratory flow between 25% and 75% of FVC

Patient numbers reflect successful performance for each parameter
