# Supplementary material for: Long-Term Burden and Respiratory Effects of Respiratory Syncytial Virus Hospitalization in Preterm Infants—The SPRING Study
Source: PLoS One. 2015 May 8;10(5):e0125422. doi: 10.1371/journal.pone.0125422 (PMC4425575; doi:10.1371/journal.pone.0125422)
Supplement: S4 Table — These antigens are the standard ones used in Spain. *χ2 test or Fisher's exact test. NS: not significant. (DOCX) [file pone.0125422.s004.docx]

**S4 Table****. Prick test results**

| **Allergen** | **Case** | **Control** | ***p**** |
| --- | --- | --- | --- |
| Cypress (tree pollen)  Positive, n/N (%) | 2/60 (3.3) | 6/167 (3.6) | NS |
| Alternaria (fungal spores)  Positive, n/N (%) | 2/62 (3.2) | 9/174 (5.2) | NS |
| Cladosporium (fungal spores)  Positive, n/N (%) | 0/57 (0.0) | 0/164 (0.0) | NS |
| D. pteronyssinus (dust mite)  Positive, n/N (%) | 3/62 (4.8) | 24/175 (13.7) | 0.042 |
| D. farinae (dust mite)  Positive, n/N (%) | 3/61 (4.9) | 19/173 (11.0) | NS |
| Cat dander  Positive, n/N (%) | 4/62 (6.5) | 2/174 (1.1) | 0.043 |
| Dog dander  Positive, n/N (%) | 1/62 (1.6) | 2/173 (1.2) | NS |

These antigens are the standard ones used in Spain

*χ^2^ test or Fisher's exact test

NS: not significant
