# Supplementary material for: Long-Term Burden and Respiratory Effects of Respiratory Syncytial Virus Hospitalization in Preterm Infants—The SPRING Study
Source: PLoS One. 2015 May 8;10(5):e0125422. doi: 10.1371/journal.pone.0125422 (PMC4425575; doi:10.1371/journal.pone.0125422)
Supplement: S5 Table — CI: confidence interval, OR: odds ratio. *χ2 test, Fisher's exact test, t-test, Mann-Whitney U test. †Mother, father and siblings. NS: not significant. (DOCX) [file pone.0125422.s005.docx]

**S5 Table****. Bivariate analysis of risk factors associated with wheezing through 6 years**

|  | **Simple Wheezing** | | **Recurrent Wheezing** | | **Severe Wheezing** | | **Total Wheezing** | |
| --- | --- | --- | --- | --- | --- | --- | --- | --- |
|  | **OR (95%CI)** | ***p*^*^** | **OR (95%CI)** | ***p*^*^** | **OR (95%CI)** | ***p*^*^** | **OR (95%CI)** | ***p*^*^** |
| **Group (Case/Control)** | 2.10  (1.37-3.23) | <0.001 | 4.87  (3.14-7.54) | <0.001 | 4.51  (2.93-6.93) | <0.001 | 3.98  (2.36-6.70) | <0.001 |
| **Sex (Male/Female)** | 1.26  (0.88-1.80) | NS | 1.00  (0.70-1.45) | NS | 1.01  (0.69-1.46) | NS | 1.42  (0.98-2.07) | 0.041 |
| **Gestational Age (32-33 weeks/34-35 weeks)** | 1.22  (0.85-1.78) | NS | 1.12  (0.77-1.63) | NS | 1.06  (0.72-1.56) | NS | 1.40  (0.96-2.05) | 0.050 |
| **Birth (Single/Multiple)** | 1.00  (0.70-1.42) | NS | 0.85  (0.59-1.22) | NS | 0.93  (0.64-1.35) | NS | 0.97  (0.67-1.40) | NS |
| **Birth weight** | **^-^** | 0.191 | **^-^** | 0.003 | **-** | 0.006 | **-** | NS |
| **Breast feeding (Yes/No)** | 0.70  (0.49-1.00) | 0.030 | 0.92  0.64-1.32) | NS | 0.83  (0.57-1.20) | NS | 0.77  (0.53-1.12) | NS |

| **Number of residents at home** | **^-^** | | NS | **^-^** | NS | **-** | NS | **-** | NS |
| --- | --- | --- | --- | --- | --- | --- | --- | --- | --- |
| **Smoking at home (Yes/No)** | 1.47  (1.03-2.11) | | 0.022 | 1.32  (0.92-1.90) | 0.079 | 1.28  (0.88-1.86) | NS | 1.60  (1.10-2.33) | 0.009 |
| **Animals in the home (Yes/No)** | 1.15  (0.75-1.77) | | NS | 0.99  (0.64-1.54) | NS | 1.17  (0.75-1.82) | NS | 1.18  (0.75-1.85) | NS |
| **Carpets in the home (Yes/No)** | | 1.60  (1.10-2.32) | 0.009 | 1.90  (1.31-2.76) | 0.001 | 1.80  (1.23-2.63) | 0.002 | 1.89  (1.27-2.81) | 0.001 |
| **Family history of atopy in first degree relative† (Yes/No)** | | 0.94  (0.65-1.34) | NS | 0.89  (0.62-1.29) | NS | 0.95  (0.65-1.37) | NS | 0.91  (0.63-1.32) | NS |
| **Children with atopy diagnosed between 0-6 years (Yes/No)** | | 1.42  (0.99-2.03) | 0.035 | 1.46  (1.01-2.10) | 0.027 | 1.74  (1.20-2.54) | 0.002 | 1.58  (1.08-2.30) | 0.011 |

CI: confidence interval, OR: odds ratio

*χ^2^ test, Fisher's exact test, t-test, Mann-Whitney *U* test

†Mother, father and siblings

NS: not significant
